# Oklahoma Territorial Medical Association

**Published:** 1901-11

**Authors:** 


COMMITTEE OF ARRANGEMENTS.

Dr. A. L. Hathcock, Chairman, Palestine, Texas; Dr. E. W. Link, Dr. E. B. Parsons.

---

### Oklahoma Territorial Medical Association.

Autumnal session, ninth year, of the Oklahoma Territorial Medical Association, at Oklahoma City, November 13, 1901, 10 a. m.

ORDER OF BUSINESS—WEDNESDAY, NOVEMBER 13, 10 A. M.

Call to order by President R. D. Love, M. D.
Invocation, Rev. L. M. Broyles, Oklahoma City.
Address of Welcome, Judge C. B. Ames, Oklahoma City.
Response, John W. Duke, M. D., Guthrie.
Roll Call.
Reading Minutes of Last Meeting.
Applications for Membership.
Report of Board of Censors.
Election of Members.
Report of Standing Committees.
New or Unfinished Business.
Report of Secretary and Treasurer.
Deciding Place of Next Meeting.

CONTRIBUTIONS.

1. Ectopic Gestation. F. P. Hulen, M. D., Pond Creek.
2. Clinical Report. A. K. West, M. D., Oklahoma City.
3. Is Gunshot Wound of the Stomach Necessarily Fatal? S. E. Knight, M. D., Enid.
4. Gonorrhea. G. A. Wall, M. D., Oklahoma City.
5. Paper. Ira G. Stone, M. D., Kingfisher.
6. Examination of Railway Employes' Eyes. John R. Hamill, M. D., Guthrie.
7. Fussy Practice. Delos Walker, M. D., Oklahoma City.
8. Paper. R. V. Smith, M. D., Guthrie.
9. Leukemia. L. N. Upjohn, M. D., Norman.
10. The Eye in Relation to General Disease. C. E. Bower, M. D., Lawton.
11. Paper. R. T. Edwards, M. D., Oklahoma City.
12. Trachoma. L. Haynes Buxton, M. D., Oklahoma City.
13. Some Practical Applications of the X-Ray in Fractures and Dislocations. J. A. Reck, M. D., Oklahoma City.
14. Paper. Herman E. Pearse, M. D., Kansas City, Mo.
15. Paper. Harry Walker, M. D., Pawhuska.
16. Report of Cases. J. B. Rolater, M. D., Oklahoma City.

OFFICERS.

President, R. D. Love, M. D., Perry.
Vice-President, John H. Scott, M. D., Shawnee.
Secretary-Treasurer, E. O. Barker, M. D., Guthrie.

BOARD OF CENSORS.

Delos Walker, M. D., Oklahoma City; J. A. Hatchett, M. D., El Reno; W. T. Salmon, M. D., Oklahoma City.

COMMITTEE OF ARRANGEMENTS.

R. T. Edwards, M. D., Oklahoma City; Lea A. Riley, M. D., Oklahoma City; C. B. Bradford, M. D., Oklahoma City.

COMMITTEE OF RECEPTION.

All members of the Association in Oklahoma City, Oklahoma.

